# The population history of *Garra orientalis* (Teleostei: Cyprinidae) using mitochondrial DNA and microsatellite data with approximate Bayesian computation

**DOI:** 10.1186/s12862-016-0645-9

**Published:** 2016-04-11

**Authors:** Jin-Quan Yang, Kui-Ching Hsu, Zhi-Zhi Liu, Li-Wei Su, Po-Hsun Kuo, Wen-Qiao Tang, Zhuo-Cheng Zhou, Dong Liu, Bao-Long Bao, Hung-Du Lin

**Affiliations:** Key Laboratory of Exploration and Utilization of Aquatic Genetic Resources, Shanghai Ocean University, Ministry of Education, 999 Huchenghuan Road, Lingang New City, Shanghai, 201306 China; Department of Industrial Management, National Taiwan University of Science and Technology, 43 Keelung Road, Section 4, Taipei, 106 Taiwan; College of Animal Sciences, Zhejiang University, Hangzhou, 310029 China; The Affiliated School of National Tainan First Senior High School, Tainan, 701 Taiwan

**Keywords:** *Garra orientalis*, Microsatellite, Mitochondria, Phylogeography, Approximate Bayesian computation

## Abstract

**Background:**

The South China landmass has been characterized by a complex geological history, including mountain lifting, climate changes, and river capture/reversal events. To determine how this complexity has influenced the landmass’s phylogeography, our study examined the phylogeography of *Garra orientalis*, a cyprinid widely distributed in South China, using sequences from the mitochondrial DNA control region and cytochrome b gene (1887 bp) and polymorphisms of thirteen microsatellite loci.

**Results:**

In total, 157 specimens were collected from eight populations. All 88 mtDNA haplotypes were identified as belonging to three major lineages, and these lineages were almost allopatric in their distributions. The results of a statistical dispersal-vicariance analysis suggested that the ancestral populations of *G. orientalis* were distributed south of the Yunkai Mountains, including on Hainan Island. The mtDNA data revealed a strong relationship between phylogeny and geography. In the microsatellite analysis, a total of 339 alleles with an average of 26 alleles per locus were observed across thirteen microsatellite loci. A clustering algorithm for microsatellite data revealed an admixture-like genetic structure. Although the mtDNA and microsatellite data sets displayed a discordant population structure, the results of an approximate Bayesian computation approach showed that these two markers revealed congruent historical signals. The population history of *G. orientalis* reflects vicariance events and dispersal related to the complex geological history of South China.

**Conclusion:**

Our results (i) found that the discordances between mtDNA and microsatellite markers were accounted for by admixtures; (ii) showed that the Wuzhishan and Yinggeling mountain ranges and Qiongzhou Strait were important barriers limiting gene exchange between populations on both sides; (iii) indicated that during glaciation and inter-glacial periods, the strait and continental shelves were exposed and sank, which contributed with the dispersion and differentiation of populations; and (iv) displayed that the admixtures between lineages took place in coastal populations and then colonized the tributaries of the Pearl River.

## Background

**Fig. 1:**
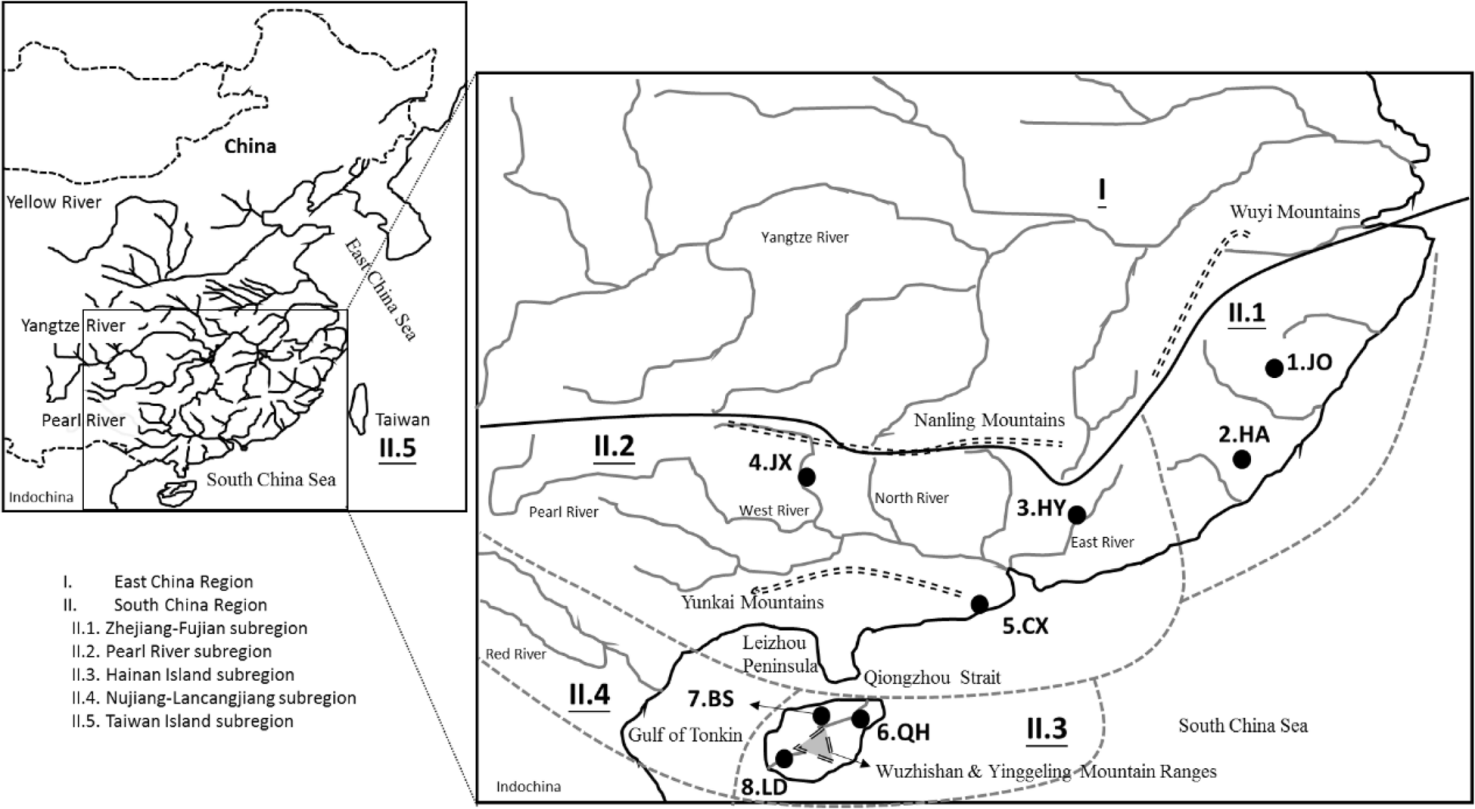
Sampling localities of *Garra orientalis* are indicated by ●. The ichthyofaunal districts were defined by Li (1981)

China, a vast geographical area with complex geology, is divided into five major geographical regions according to the essential geo-historical events and ichthyofauna [1]. These regions are as follows: (1) North China, (2) West China, (3) Mongolia–Ningxia, (4) East China and (5) South China. Among these five regions, South China, located south of the Yangtze River (excluding the river itself) and east of the Wuyi Mountains, is divided into five subregions: Taiwan Island, Zhejiang-Fujian, Pearl River, Nujiang-Lancangjiang and Hainan Island (Fig. 1).

The Zhejiang-Fujian subregion, including the southeastern coastal districts, is located in southeastern China, south of the Yangtze River, north of the Pearl River, and east of the Wuyi Mountains. The southeastern coastal districts are geographically separated from the Yangtze and Pearl Rivers. According to geological history and biogeographic studies [2–4], the southeastern coastal districts had not been formed until the Pliocene (ca. 2 mya), and in the mid-Pleistocene, the rising of the Wuyi Mountains hindered the inter-river migrations between the Yangtze River and southeastern coastal districts. A study of the evolutionary history of *Cobitis sinensis* (Sauvage & Dabry de Thiersant, 1874) supported this hypothesis [5].

The Pearl River subregion includes two parts: the Pearl River and the Leizhou Peninsula (south of the Yunkai Mountains). The Pearl River or Zhujiang, the largest river in South China, is composed of three major tributaries: the West, North and East Rivers. Chen et al. [6] studied the developmental history of the Pearl River Delta and found that primary freshwater fish in different tributaries of the Pearl River had little contact with each other, and the similarities among the fish fauna were likely due to confluences during several ice ages in the Pleistocene. Based on the composition of the fish fauna, Chen et al. [6] suggested separating three major tributaries of Pearl River into two independent rivers: the West and North Rivers and the East River. In addition, geological records [3] suggest that the East River once flowed northwards to southeastern coastal districts, whereas it currently flows southward to the Pearl River. Chiang et al. [5] suggested that the *C. sinensis* populations in the East River were closer to those in the southeastern coastal districts than in other tributaries of the Pearl River. Moreover, the rivers on the northern side of the Yunkai Mountains flow northward to the Pearl River, and those on the southern side flow southward to the Gulf of Tonkin and Qiongzhou Strait. Chen et al. [7] examined the phylogeography of *Glyptothorax* in East Asia, and found that the two species, *G. hainanensis* and *G. fokiensis*, were restricted to the regions south and north of the Yunkai Mountains, respectively.

The Nujiang-Lancangjiang subregion is a specific geographical region. All rivers, including the Yuanjiang (upstream of the Red River), Lancangjiang (upstream of the Mekong River), and Nujiang (upstream of the Salween River), drain into Indochina. Clark et al. [8] proposed that the Upper Yangtze, Middle Yangtze, Upper Mekong, and Upper Salween Rivers once drained into the South China Sea through the Red River. Peng et al. [9] and Guo et al. [10] also supported this evolutionary history of the river system based on the phylogenetic relationships of sisorid catfishes.

Taiwan and Hainan Island are the first and second largest islands in mainland China. During the glaciations, the continental shelves and land bridges in the Taiwan Strait, Qiongzhou Strait and Gulf of Tonkin were exposed by dropping sea levels, which assisted in the migration of biota. It has been documented that Taiwan and Hainan Island were parts of the Asian continent during the past million years [5, 7, 11–18]. In addition to geological evidence, biological studies indicate a close evolutionary relationship between Taiwanese and Chinese continental species [5, 18–23]. Moreover, during the middle Pleistocene, the whole region, including the Leizhou Peninsula, Qiongzhou Strait, Gulf of Tonkin and Hainan Island, became a part of the coastal plain of the Asian continent, and all rivers in the southern region of the Yunkai Mountains and North Vietnam (i.e., the Red River) drained through the Leizhou Peninsula and Hainan Island into the South China Sea. Geological evidence and biological studies indicate a close evolutionary relationship among populations in the southern region of the Yunkai Mountains, on Hainan Island, and in North Vietnam [7, 24, 25].

To determine how this complexity has influenced phylogeography, our study examines the phylogeography of freshwater fish because they are restricted to river systems and therefore provide excellent opportunities for testing the influences of geological events on the distribution of taxa and genetic variations [26, 27]. The oriental sucking barb, *Garra orientalis* Nichols, 1925 (Labeoninae), is a small- to moderate-sized freshwater fish in the Cyprinidae family. This species lives in rapids and attaches to rocks using a sucking disc [28]. This species is widely distributed in South China, including the Zhejiang-Fujian (i.e., in the Hanjiang and Minjiang Rivers, southeastern coastal districts), Pearl River, Nujiang-Lancangjiang (i.e., in the Yuanjiang and Lancang Rivers) and Hainan Island subregions (Fig. 1) [29]. According to this distribution pattern, *G. orientalis* is an ideal fish species to study the biological consequences of the complex geological history of river systems in South China. Moreover, it is widely acknowledged that nuclear and mitochondrial markers react differently to current demography as well as to past history, so the use of both types of markers is advocated to gain insight into both historical and contemporary processes. Thus, this study used two different biologically characterized genetic markers, mitochondrial DNA sequences and microsatellite polymorphisms, to establish the phylogeographic pattern of *G. orientalis* in South China. There are three major questions in our study: (1) Do these two genetic markers reveal congruent signals of population history? (2) How and when did *G. orientalis* colonize the rivers of different geographical districts in South China? (3) Is there a phylogeographic break in the freshwater fish of South China?

## Results

### Mitochondrial DNA diversity

A total of 65 D-loop haplotypes (751 bp, 48 phylogenetically informative sites: KR698422–KR698486) and 31 cytochrome b (cyt b) haplotypes (1136 bp, 12 phylogenetically informative sites; KR698391–KR698421) were obtained for 157 *G. orientalis* specimens from the eight populations analyzed (Table 1; Fig. 1). We obtained both fragments for all 157 fish and used both fragments of mtDNA to examine the population structure. In total, 88 mtDNA haplotypes were defined by 110 variable sites and 60 phylogenetically informative sites. Nucleotide sequences were A + T (62.5 %) rich. The mean haplotype diversity in each population was 0.981 (range: 0.801 to 1.00). The haplotype diversity in each population within the Pearl River subregion (0.980–1.000) was higher than that within other subregions (0.816–0.859 for Zhejiang-Fujian and 0.801–0.953 for Hainan Island). At the subregion level, the Pearl River subregion showed the highest haplotype diversity (0.986). However, the sample sizes in the Pearl River subregion and in each population within the Pearl River subregion were not larger than other subregions and populations (Table 1). These results reveal that genetic diversity was not correlated with sample size. Thus, our study suggests that although the sample size of 9 individuals from population Heyuan (HY) is small, the number of samples is sufficient. Estimates of the current (θ_π_) and historical (θ_ω_) genetic diversity for each sample indicated that all populations showed a pattern of decline (θ_π_ < θ_ω_). At the subregion level, only the Hainan Island subregion displayed a pattern of growth (θ_π_ > θ_ω_). The genetic diversity indicated shrinking local populations and high divergence rates on Hainan Island.

**Table 1:** Sampling locations, codes, sample size (mitochondrial/microsatellite), haplotype and nucleotide diversity of mtDNA and microsatellite diversity indices. Average number alleles/locus (A), mean allelic richness (A_R_) per population, expected (H_E_) and observed (H_O_) heterozygosities

Locations (Abbreviation)	Longitude	Latitude	Sample size	Mitochondrial DNA							
				Haplotype diversity (h)	Nucleotide diversity		Microsatellite loci				
					θ_π_ (%)	θ_ω_ (%)	A	A_R_	H_O_	H_E_	F_IS_
Zhejiang-Fujian subregion			33/33	0.835	0.122	0.196	9.308	7.029			
1.Jian’ou (JO)	118.18	27.02	13/13	0.859	0.075	0.085	7.385	6.283	0.905	0.790	−0.153
2.Huaan (HA)	117.31	25.00	20/20	0.816	0.134	0.179	11.231	7.774	0.896	0.863	−0.039
Pearl River subregion			52/52	0.986	0.391	0.762	12.128	8.587			
3.Heyuan (HY)	114.41	23.44	9/9	1.000	0.384	0.565	8.154	7.697	0.755	0.851	0.119
4.Jinxiu (JX)	110.10	24.07	23/23	0.980	0.333	0.657	14.308	9.085	0.919	0.905	−0.016
5.Chunxi (CX)	111.56	22.27	20/20	0.989	0.468	0.488	13.923	8.980	0.732	0.900	0.191
Hainan Island subregion			72/71	0.960	0.777	0.689	13.461	8.271			
6.Qionghai (QH)	110.18	19.09	24/23	0.953	0.250	0.284	14.385	8.704	0.886	0.872	−0.016
7.Basha (BS)	109.26	19.13	24/24	0.920	0.197	0.284	15.077	9.056	0.922	0.885	−0.044
8.Ledong (LD)	109.10	18.44	24/24	0.801	0.257	0.312	10.923	7.054	0.767	0.780	0.041
Total			157/156	0.981	0.744	1.064	11.923	10.001			

Among the 88 haplotypes, only ten haplotypes (G1–G10) were shared between more than two populations (Table 2). The most widespread haplotype was G3, distributed among four populations (HA, HY, JX and CX). The haplotypes G7–G10 were only distributed in two Hainan Island populations, QH and BS. Among the eight sampling populations, seven populations had more than two shared haplotypes, and only population LD did not have any shared haplotypes. The average pairwise F_ST_ (Table 3) within the Zhejiang-Fujian, Pearl River and Hainan Island subregions were 0.16, 0.01 and 0.56, respectively, and the pairwise F_ST_ among these three subregions was 0.55, with a range from 0.18 to 0.81. A comparison of the fixation indices N_ST_ and G_ST_ revealed that N_ST_ was much larger than G_ST_ (0.651 and 0.072, respectively). This result suggested a strong relationship between phylogeny and geography. These results showed that the population differentiations were significant.

**Table 2:** The distribution information of the shared mtDNA haplotypes and alleles and private alleles of 13 microsatellite loci. MT indicates the mtDNA lineages in Fig. 2. S and P indicate the number of shared and private haplotypes

		JO	HA	HY	JX	CX	QH	BS	LD
MtDNA									
G1		4	8	1	0	0	0	0	0
G2		3	3	0	0	0	0	0	0
G3		0	3	1	1	1	0	0	0
G4		0	1	0	1	2	0	0	0
G5		0	0	1	1	0	0	0	0
G6		0	0	1	1	2	0	0	0
G7		0	0	0	0	0	1	1	0
G8		0	0	0	0	0	1	7	0
G9		0	0	0	0	0	2	1	0
G10		0	0	0	0	0	3	1	0
S		2	4	4	4	3	4	4	0
P		4	4	5	15	15	11	13	11
MT		I	I	I,II	I,II	I,II	II	II	III
Microsatellite									
Gar1	29	5	8	8	12	20	13	9	3
Gar2	18	9	11	6	14	11	13	15	11
Gar3	21	3	10	7	11	10	13	8	7
Gar4	32	8	12	6	14	16	21	20	14
Gar5	27	5	10	9	14	10	9	14	12
Gar6	30	15	14	14	20	17	19	21	15
Gar7	23	8	12	7	13	15	10	16	11
Gar8	28	7	13	4	15	12	8	17	10
Gar9	35	8	10	7	16	12	25	17	9
Gar10	29	6	9	12	14	16	17	22	15
Gar11	24	5	13	5	11	14	14	14	14
Gar12	23	9	14	13	15	13	14	12	10
Gar13	20	7	9	7	15	14	10	11	11
total	339	95	145	105	184	180	186	196	142
Private		0	5	1	7	25	19	9	3

**Table 3:** Matrix of pairwise F_ST_ based on mtDNA (above diagonal) and microsatellite (below diagonal) data. Refer to Table 1 for the abbreviations of localities

	JO	HA	HY	JX	CX	QH	BS	LD
JO		0.16	0.24	0.26	0.26	0.79	0.82	0.85
HA	0.02		0.09	0.09	0.13	0.76	0.80	0.84
HY	0.09	0.07		0.03	0.01	0.63	0.67	0.77
JX	0.03	0.02	0.04		0.00	0.64	0.66	0.78
CX	0.12	0.08	0.08	0.05		0.56	0.58	0.74
QH	0.12	0.09	0.08	0.06	0.06		0.01	0.82
BS	0.08	0.06	0.06	0.03	0.06	0.07		0.84
LD	0.12	0.09	0.10	0.07	0.12	0.12	0.06	

**Fig. 2:**
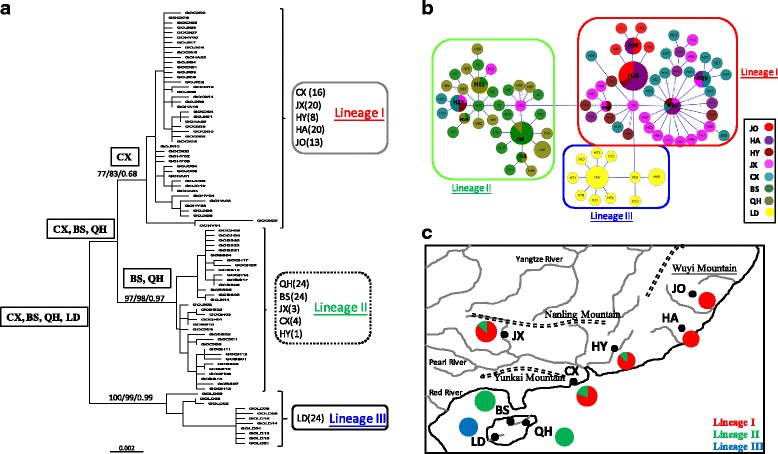
Phylogenetic analysis based on mitochondrial DNA cytochrome b and D-loop sequences. **a** The ML tree with HKY model. The numbers at the nodes are bootstrap values of the ML, NJ and BI analyses. The haplotype network **b** and the distribution of the major mtDNA lineages **c**

In phylogenetic analyses, haplotype trees reconstructed with different methods (i.e., ML, NJ and BI, rooted and unrooted) were identical, with only small differences in bootstrap values. In the ML tree (Fig. 2a), 88 haplotypes fell into three major lineages (I-III) with significant bootstrap support. However, the relationships among these three lineages was not supported by bootstrap analysis in all phylogenetic trees. Thus, our study used a haplotype network in Arlequin to determine the relationships among these three lineages. The network (Fig. 2b) also supported the notion that all mtDNA haplotypes fell into three major lineages (I-III), with lineage I located at the interior and the others located at the tip. Lineage I included five populations in the Zhejiang-Fujian and Pearl River subregions; lineage II contained three populations in the Pearl River subregion and two populations in the northern part of Hainan Island (BS and QH); lineage III was only distributed in population LD in the southern region Hainan Island (Fig. 2c). Lineage III was allopatric to lineages I and II, and lineages I and II were sympatric in the Pearl River subregion. The genetic distances among these three lineages ranged from 0.009 to 0.015 (mean = 0.013), and the mean divergences within lineages was 0.002 (ranging from 0.002 to 0.003). The results of a BEAST analysis suggested that the time to coalescence for *G. orientalis* was estimated to be at some time in the Pleistocene (T_MRCA_ = 0.462 mya, 0.343–0.591). The T_MRCA_ of the three major phylogroups (lineages I–III) were 0.367 (0.265–0.484), 0.370 (0.265–0.492) and 0.458 (0.331–0.591), respectively.

The results of AMOVA analysis indicated significant genetic structures at several levels (Table 4). The partitioning of genetic diversity based on three subregions or the mainland and island (schemes 1 and 2) revealed that only a small portion of the genetic variability was accounted for by differences between the geographic districts (23.73 and 35.72 %, respectively). The results also showed that only a small portion of the variability was accounted for by differences between the Zhejiang-Fujian and Pearl River subregions. Moreover, our study divided these eight populations of *G. orientalis* into three groups (scheme 3) based on the distribution of shared haplotypes (Table 2). We found that the majority of the variability was accounted for by between-group differences among these three groups, Zhejiang-Fujian + Pearl River, QH + BS, LD (72.89 %). After comparing schemes 3 and 4, the results supported two phylogeographic breaks, one at the Qiongzhou Strait and another caused by the Wuzhishan and Yinggeling Mountain Ranges (WY Ranges) on Hainan Island (Fig. 1). Moreover, compared with schemes 3 and 5, the results showed the most genetic variability was not distributed among clustering lineages.

**Table 4:** Analysis of molecular variance (AMOVA) of *Garra orientalis* based on mtDNA data

Scheme	Category description	% Var.	Statistic	*p*
1. Three geographical groups (Zhejiang-Fujian) (Pearl River) (Hainan Island)				
	Among regions	23.73	F_SC_ = 0.61	<0.001
	Among populations in region	46.24	F_ST_ = 0.70	<0.001
	Within population	30.03	F_CT_ = 0.24	<0.001
2. Two geographical groups (Zhejiang-Fujian + Pearl River) (Hainan Island)				
	Among regions	35.72	F_SC_ = 0.58	<0.001
	Among populations in region	37.11	F_ST_ = 0.73	<0.001
	Within population	27.17	F_CT_ = 0.36	<0.001
3. Three groups based on the distribution of shared haplotypes in Table 2 (Zhejiang-Fujian + Pearl River) (QH + BS) (LD)				
	Among regions	72.89	F_SC_ = 0.09	<0.001
	Among populations in region	2.5	F_ST_ = 0.75	<0.001
	Within population	24.61	F_CT_ = 0.73	<0.001
4. Two groups (Zhejiang-Fujian + Pearl River + QH + BS) (LD)				
	Among regions	56.68	F_SC_ = 0.55	<0.001
	Among populations in region	24.00	F_ST_ = 0.81	<0.001
	Within population	19.32	F_CT_ = 0.57	<0.001
5. Four groups based on the distribution of lineages (Zhejiang-Fujian) (Pearl River) (QH + BS) (LD)				
	Among regions	71.06	F_SC_ = 0.02	<0.1
	Among populations in region	0.48	F_ST_ = 0.72	<0.001
	Within population	28.46	F_CT_ = 0.71	<0.001

The results of the S-DIVA analysis produced a scenario with vicariance and dispersion events that shaped the current distribution patterns of *G. orientalis* (Fig. 2). This analysis indicated four possible ancestral populations for *G. orientalis*, all of which were widespread in the southern region of the Yunkai Mountains. Two vicariance events, the rising of the WY Ranges on Hainan Island (Fig. 1) and the formation of the Qiongzhou Strait, separated ancestral populations of *G. orientalis* into three lineages, I-III. After the division of the ancestral areas, lineage II reached the Pearl River subregion, and lineage I reached the Zhejiang-Fujian subregion by dispersal events. Based on the results of S-DIVA and phylogenetic analysis (Fig. 2), *G. orientalis* seemed to originate from Hainan Island and move northward to the mainland.

The IM program was used to calculate the amount and directions of migration between the Hainan Island and Pearl River subregions and between the Pearl River and Zhejiang-Fujian subregions. The migration rates from Zhejiang-Fujian to Pearl River (m_ZP_ = 0.685, 0.040–0.976 within lineage I only; m_ZP_ = 0.673, 0.044–0.977 within all data) were higher than those from Pearl River to Zhejiang-Fujian (m_PZ_ = 0.061, 0.014–0.875 within lineage I only; m_PZ_ = 0.069, 0.013–0.924 within all data), while migration rates in both directions between the Hainan Island and Pearl River subregions (m_HP_ and m_PH_) were shown to be near zero.

### Microsatellite DNA variations

A total of 339 alleles with an average of 26 alleles per locus were observed across thirteen microsatellite loci, ranging from 18 (Gar2) to 35 alleles (Gar9) (Table 2). Among the 339 alleles, there were 15 shared alleles from eight loci among these eight populations, and 69 alleles were private. Populations CX and QH had the most private alleles (Table 2). The estimates of genetic variability are summarized for each population in Table 1. Across all populations, the average number of alleles per locus (A) was 11.923, the average allelic richness (A_R_) was 10.001 and the average observed (Ho) and expected heterozygosity (H_E_) were 0.848 and 0.856, respectively (Table 1). The genetic heterozygosity was relatively high. Population JO (A = 7.385, A_R_ = 6.283; Zhejiang-Fujian subregion), population HY (A = 8.154, A_R_ = 7.697; Pearl River subregion) and population LD (A = 10.923, A_R_ = 7.054; Hainan Island subregion) showed lower genetic diversity. Moreover, population HA in the Zhejiang-Fujian subregion also displayed a lower level of genetic diversity (A = 11.231, A_R_ = 7.774). Other populations (JX, CX, QH and BS) displayed similar levels of genetic diversity (A = 13.923–15.077, A_R_ = 8.980–9.056). At the subregion level, the Hainan Island subregion showed the highest average number of alleles per locus (13.461) (Table 1). The inbreeding coefficients (*F*_IS_) of most populations were negative and ranged from −0.153 (JO) to 0.191 (CX). The index (*F*_IS_) did not deviate significantly from zero at any locus in any population, suggesting that HWE could be assumed in all populations (Table 1).

**Fig. 3:**
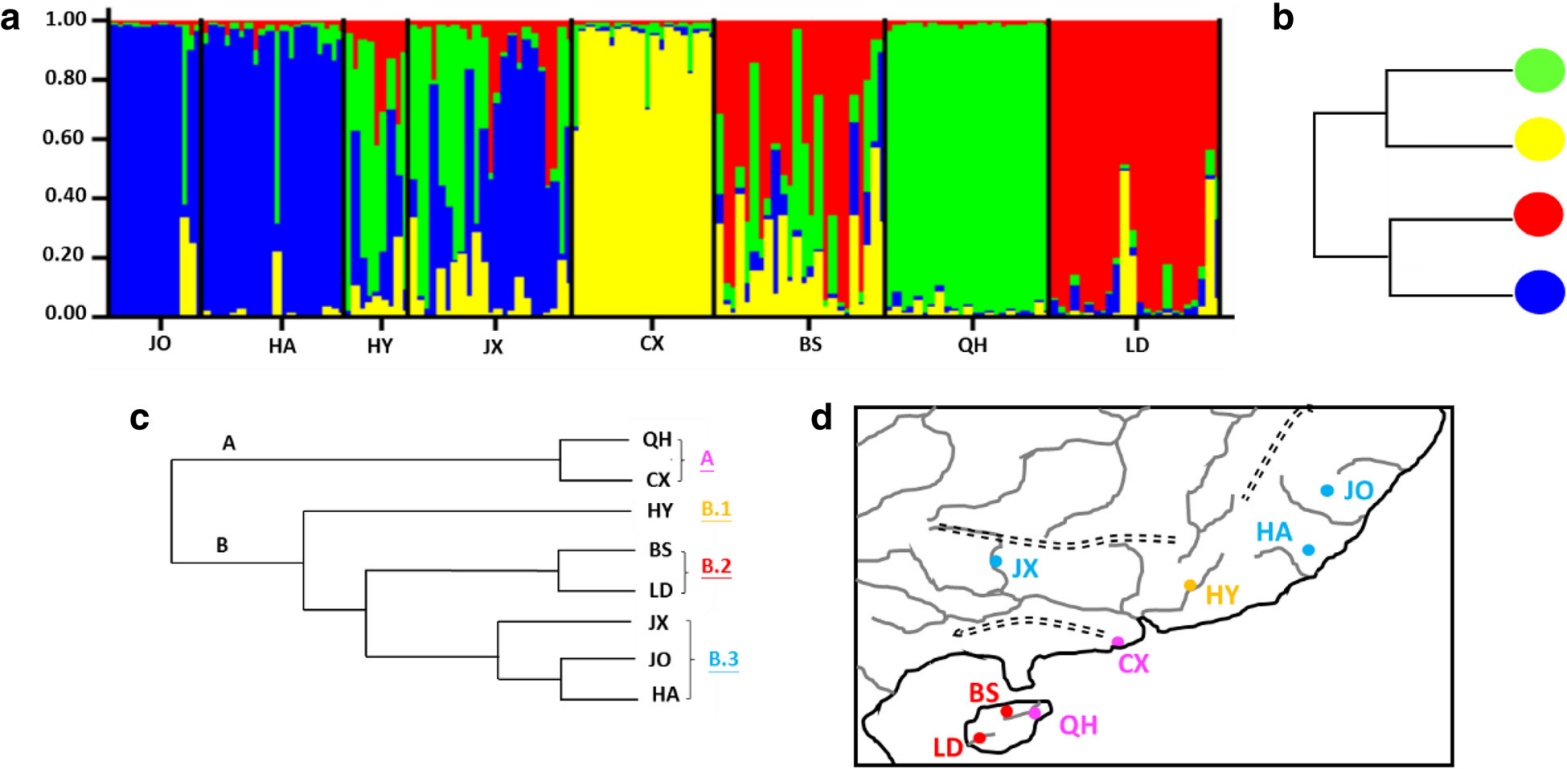
STRUCTRUE and NJ trees based on microsatellite data. **a** The frequency of inferred population clusters with the program STRUCTURE. Membership coefficients inferred for K = 4. Each of the inferred population clusters is represented by a color. **b** The NJ tree, visualizing genetic distances among the inferred clusters. **c** The NJ tree of genetic relationships among eight populations using D_A_ genetic distance. **d** The distribution of groups revealed in the NJ tree

Analyses of microsatellite data using the STRUCTURE clustering algorithm indicated the presence of four distinct genetic clusters (K = 4). Population level admixture analysis indicated that two populations (HY and JX) contained all four clusters, the population BS included three clusters, and others only contained one cluster (Fig. 3a). Moreover, the NJ tree constructed by visualizing genetic distances among the inferred clusters (Fig. 3b) showed that these four clusters were identified as two groups. The neighbor joining phylogenetic tree of genetic relationships among eight populations (Fig. 3c) constructed from D_A_ genetic distances revealed that two main groups were identified; the first group (group A) included populations QH and CX (more private alleles, Table 2), and the second group (group B) contained populations HY, BS, LD, JX, JO and HA. The second group was separated into three subgroups (B.1–B.3). Our study suggests that the microsatellite groups were present across the geological barriers. For example, group A was distributed on both sides of the Qiongzhou Strait (Fig. 3d). Geographical division assessed by GENEPOP (Table 3) indicated lower genetic differentiation among populations (with F_ST_ ranging from 0.02 to 0.12). The average pairwise F_ST_ values within the Zhejiang-Fujian, Pearl River and Hainan Island subregions were 0.02, 0.06 and 0.08, respectively, and the F_ST_ among these three subregions was 0.08, with a range from 0.07 to 0.09.

### Population history in DIYABC

**Fig. 4:**
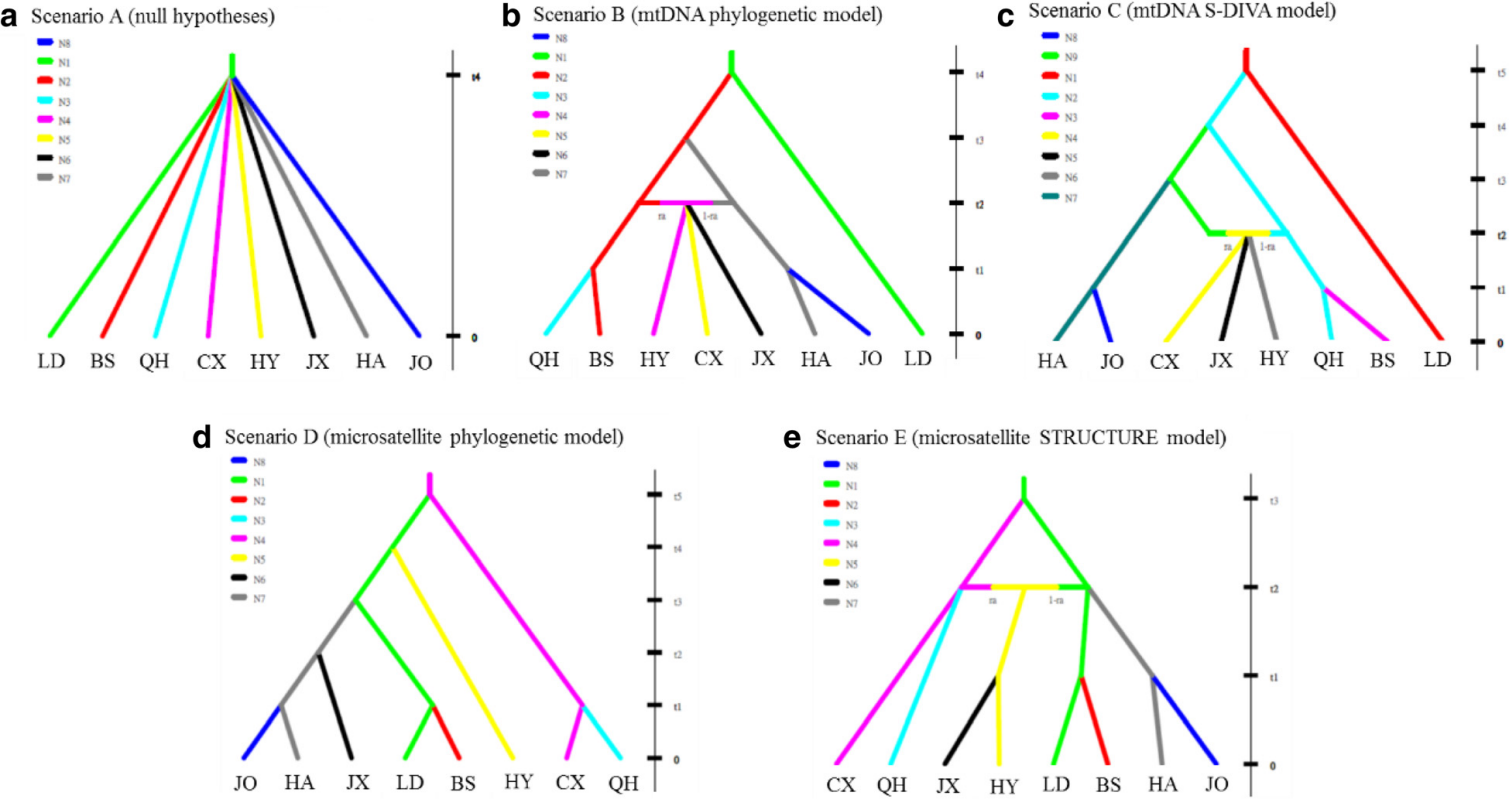
Graphical representation of the five scenarios, **a** null hypotheses, **b** mtDNA phylogenetic model, **c** mtDNA S-DIVA model, **d** microsatellite phylogenetic model, and **e** microsatellite STRUCTURE model, used in the ABC analyses. N values are population sizes, and t values correspond to the timing of past divergence events or past admixture between populations. Note that time is not to scale

To understand the population history of *G. orientalis*, our study used three types of data to examine five population history scenarios with the DIYABC program (Fig. 4, see Methods: *Population history*). For the mtDNA data set, the highest posterior probability was found for scenario C (mtDNA S-DIVA model, Fig. 4c). Its posterior probability (0.9058, 95 % CI: 0.8934–0.9181) was much higher than for other scenarios (Fig. 4, Table 5). The 95 % CI of scenario C did not overlap with those for other scenarios (Table 5). The posterior probability of scenario B (mtDNA phylogenetic model, Fig. 4b) was much lower than for scenario C. These results suggested that the results of S-DIVA were supported by the DIYABC analysis. For the microsatellite data set, although scenario E (microsatellite STRUCTURE model, Fig. 4e) was expected to be the most likely, the highest posterior probability was found for scenario B, and its posterior probability (0.3585, 95 % CI: 0.3333–0.3837) was higher than for the other scenarios (Table 5). For the combined mtDNA and microsatellite data sets, the highest posterior probability was found for scenario C (0.5555, 95 % CI: 0.5227–0.5882). Moreover, the posterior probability of scenario B (0.4432, 95 % CI: 0.4104–0.4759) was less than scenario C, and the 95 % CIs of these two scenarios did not overlap. Accordingly, our study suggests that the mtDNA and microsatellite data sets supported scenarios B and C, respectively. However, these two scenarios (B and C) were both reconstructed by mtDNA data (for a discussion of the difference between these two scenarios, see Discussion: *Phylogeography of Garra orientalis*). Thus, our study finds that congruent signals of population history were revealed by mtDNA and microsatellite.

**Table 5:** Relative posterior probabilities for each scenario (Fig. 4) and their 95 % confidence intervals based on the logistic estimate by DIYABC

Scenario	Posterior probability	95 % CI (lower-upper)
Mitochondrial DNA cyt b and D-loop genes		
Scenario A	0.0000	0.0000–0.0013
Scenario B	0.0942	0.0819–0.1066
Scenario C	0.9058	0.8934–0.9181
Scenario D	0.0000	0.0000–0.0013
Scenario E	0.0000	0.0000–0.0013
Thirteen microsatellite loci		
Scenario A	0.1286	0.1022–0.1549
Scenario B	0.3585	0.3333–0.3837
Scenario C	0.2010	0.1825–0.2195
Scenario D	0.0412	0.0000–0.0911
Scenario E	0.2708	0.2485–0.2930
Mitochondrial and microsatellite		
Scenario A	0.0000	0.0000–0.0261
Scenario B	0.4432	0.4104–0.4759
Scenario C	0.5555	0.5227–0.5882
Scenario D	0.0004	0.0000–0.0265
Scenario E	0.0009	0.0000–0.0269

## Discussion

### Incomplete lineage sorting vs. admixture

According to the spatial genetic structure of *G. orientalis* (Figs 2 and 3), our study found that the distributions of mtDNA lineages were restricted by geological barriers (Fig. 2), but the microsatellite clusters were not (Fig. 3). Moreover, the mtDNA data showed a strong relationship between phylogeny and geography (N_ST_ > G_ST_), and microsatellite data did not (Table 3). Thus, our study suggests that these two genetic markers reveal a discordant structure, and the phylogeography of mtDNA showed more structure than that of microsatellite data. According to previous studies [30–34], the discordance between structures generated with mtDNA and microsatellite data could simply be due to incomplete lineage sorting of ancestral polymorphisms, differences between male and female dispersal rates, and recent admixture. However, there were no studies to support male- or female-biased dispersal in primary freshwater fish. Moreover, lineage sorting has been the most frequent explanation for such discordance, although it is difficult to differentiate between incomplete lineage sorting and admixture. Qu et al. [34] considered that, compared with mtDNA, microsatellite loci have a higher mutation rate [35] and therefore are expected to show a geographical pattern similar to that of mtDNA. Thus, if admixture between the lineages did not take place, the mtDNA and microsatellite data would show similar divergence patterns. Alternatively, if the differentiation between mtDNA and microsatellites is the result of admixture, microsatellite and nuclear DNA (nDNA) sequence data sets would display a similar divergence pattern [34, 36].

In this study, the microsatellite loci examined were cloned from total DNA [37]. Our study also checked the complete mitochondrial genomes of *G. orientalis* (JX290078) and found the microsatellite loci in this study were not located in mitochondrial genomes. Thus, the microsatellite loci examined in our study definitely assorted with nDNA. Zink and Barrowclough [38] proposed that mtDNA lineage sorting should be completed before sorting of nDNA due to differences in effective population size. Crochet [39] suggested that similar values between the corrected mtDNA genetic differentiation [F_ST_(mt)] and their analogue F_ST_(nuc) for nuclear genes were expected. Furthermore, Brito [40] proposed an equation, F_ST_(nuc) = F_ST_(mt)/[4–3 F_ST_(mt)], to correct F_ST_ differentiation between mtDNA and nDNA. In our study, we obtain a corrected F_ST_(mt) equal to 0.32, which is still four times larger than F_ST_ value calculated with microsatellite data (0.07). Accordingly, the differences in effective population size between mtDNA and microsatellite markers cannot account for the discordant patterns present. Thus, we suggested that incongruent genetic structures between these two genetic markers might have resulted from recent admixture events.

### Approximate Bayesian computation

To reconstruct the unknown history of *G. orientalis* and test whether the mtDNA and microsatellites showed the same signals of population history, our study included approximate Bayesian computations (ABC) on our mtDNA and microsatellite data sets using the software DIYABC. Moreover, our study displayed admixture-like genetic structures of *G. orientalis* (Figs 2 and 3). Prior studies [41, 42] demonstrated that an admixture-like genetic structure detected by clustering methods (e.g., STRUCTURE analysis) is the result of simple population splitting, not admixing, using an ABC analysis of empirical and simulated data sets. Therefore, if we discuss admixture or secondary contact on the basis of clustering methods without reference to coalescent-based analysis, we will incorrectly infer the population history.

The first aim of DIYABC analysis is to examine whether the mtDNA and microsatellites showed the same signals of population history. Our results of DIYABC analyses (Table 5) showed that the microsatellite data set supported the mtDNA phylogenetic model (scenario B). Although the contemporary patterns of diversity were different between these two markers (Figs 2 and 3), our study found that congruent signals of population history were revealed by microsatellite and mtDNA markers (Fig. 4, Table 5).

The second aim of the DIYABC analysis was to reconstruct the population history of *G. orientalis*. In the DIYABC results (Table 5), two scenarios, B and C, were supported. These two supported scenarios (B and C) both displayed admixed populations in the Pearl River subregion (CX, JX and HY); the difference between these two scenarios was in the ancestral populations of the admixed populations. In scenario B (Fig. 4b), the ancestral populations of lineages I and II colonized these three populations (HY, CX and JX) in the Pearl River subregion, simultaneously; in scenario C (Fig. 4c), lineages I and II admixed in the coastal population CX and then migrated to lower and upper tributaries of the Pearl River. If scenario B was accepted, we would detect migrations between the Hainan Island and Pearl River subregions and between the Zhejiang-Fujian and Pearl River subregions. However, the results of IM analysis showed that significant migrations only existed in one direction, from Zhejiang-Fujian to the Pearl River. The migration rate between the Hainan Island and Pearl River subregions was zero. Moreover, the landforms also did not support the hypothesis that the ancestral populations of lineage II colonized the tributaries of the Pearl River, especially of the upper streams, directly from the northern region of Hainan Island. Thus, our study suggests that scenario C (mtDNA S-DIVA model) could explain the population history of *G. orientalis*. In addition, these results demonstrated an admixture-like genetic structure of *G. orientalis*, which resulted in the incongruent genetic structures between the two genetic markers.

### Phylogeographic break

According to the frequencies of these three mtDNA lineages in each population (Fig. 2c), our study divided the eight *G. orientalis* populations into four groups, Zhejiang-Fujian (lineage I), Pearl River (lineage I + II), northern Hainan Island (lineage II), and southern Hainan Island (lineage III). Based on this grouping scheme, our study proposed three barriers: the central mountainous area of Hainan Island (WY Ranges), Qiongzhou Strait, and Hanjiang River (boundary between Zhejiang-Fujian and Pearl River subregions) (Fig. 1). Moreover, based on the distribution information of the shared mtDNA haplotypes (Table 2), haplotypes G1–G6 were only distributed in the Zhejiang-Fujian and Pearl River subregions (north of Qiongzhou Strait), haplotypes G7–G10 were only distributed at populations BS and QH (north of WY Ranges and south of Qiongzhou Strait), and population LD (southern Hainan Island) did not include any shared mtDNA haplotypes (south of WY Ranges). Furthermore, based on the pairwise F_ST_ among populations, only population LD had high genetic differentiation among populations within a subregion (Table 3). Based on these results (Fig. 2; Tables 2 and 3), our study proposed three possible phylogeographic breaks: the WY Ranges, Qiongzhou Strait, and Hanjiang River.

Based on the three breaks described above, the AMOVA analysis (Table 4) indicated that most of the total genetic variance was not distributed among the four groups based on the distribution of mtDNA lineages (scheme 5). In a comparison of schemes 1 and 2, and schemes 3 and 5, the result supported that genetic variations were not significantly distributed between the Zhejiang-Fujian and Pearl River subregions, and the Qiongzhou Strait was a more effective barrier than the Hanjiang River. The results of an AMOVA showed that most of the total genetic variances were distributed among three groups, Zhejiang-Fujian + Pearl River subregions, QH + BS and LD (scheme 3). Herein, two phylogeographic breaks, the WY Ranges and Qiongzhou Strait, were supported.

### Phylogeography of *Garra orientalis*

In conclusion, our study showed that the patterns of genetic diversity differed between mtDNA and microsatellite sequences, but these two data sets revealed congruent signals of population history based on a DIYABC analysis. Our results suggested the mtDNA S-DIVA model (Fig. 4c) can explain the population history of *G. orientalis*. Moreover, estimating the time of evolutionary events is an important process for understanding the evolutionary forces that influence phylogeographic patterns [43]. This study used a divergence rate of 2.00 % per million years previously postulated by Brown et al. [44] and Bermingham and Martin [45] to calibrate the times of evolutionary events in *G. orientalis*. The T_MRCA_ estimated based on the mtDNA cyt b and D-loop sequences suggest that the origin of *G. orientalis* can be traced back to the late Pleistocene (0.462 mya). Moreover, *G. orientalis* is distributed in South China, south of the Nanling Mountains and east of the Wuyi Mountains. According to geological history [2–5], the Wuyi Mountains arose in the mid-Pleistocene. Therefore, this molecular clock likely provides correct estimates.

**Fig. 5:**
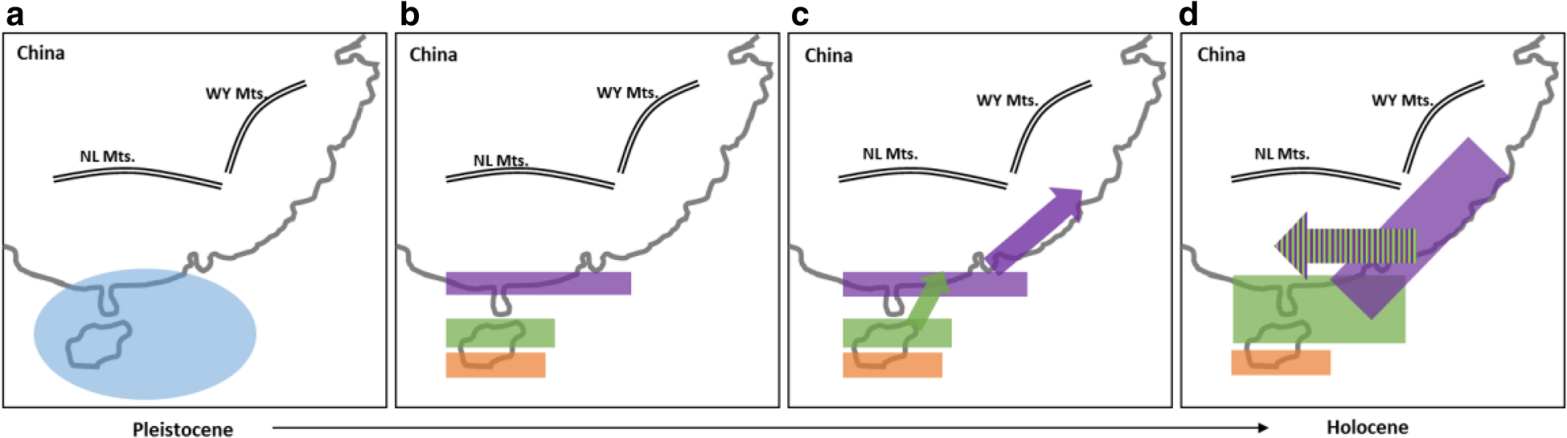
The colonization history of *Garra orientalis*. The blue area indicates the distribution of ancestral populations **a**, and **b**-**c** the orange, green and purple are the distributions of mtDNA lineages I-III in Fig. 2, respectively. **d** The lineages I(purple) and II (green) were admixed in coastal populations and then migrated from coastral populations to lower and upper tributaries of the Pearl River

Based on all the results presented here, our study suggests that the ancestral populations of *G. orientalis* were distributed widely in the southern Yunkai Mountains, including the Leizhou Peninsula and Hainan Island, in the late Pleistocene (Fig. 5a). Previous biogeography studies [17, 24] supported that during the Pleistocene glaciations, the whole region of the Gulf of Tonkin and the Qiongzhou Strait became part of the coastal plain of the Asian continent. Thus, the gene flows among the ancestral populations (CX, BS, QH and LD) were unlimited. Subsequently, our results (Figs 2 and 4c) suggested that the population on southern Hainan Island (LD) diverged by a vicariance event. Our study suggested that at ca. 0.458 mya, the WY Ranges arose on Hainan Island and isolated the population in the southern Hainan Island (LD) region as lineage III (Fig. 5b). The WY Ranges are located in the central and southern regions of Hainan Island and approach an elevation of 1800 m. Lin et al. [46] found the WY Ranges were an important barrier limiting gene exchange between populations on both sides of this mountain based on the phylogeography of Reeves’s butterfly lizard (*Leiolepis reevesii*). The landforms reflect the fact that the rivers on Hainan Island originate from the central mountainous area (including the WY Ranges) and flow outwards. The rivers in the northern region of the WY Ranges flow northward, and the rivers in the southern region of the WY Ranges flow southward. In our study, the directions of water flow in populations BS and QH are northward into Qingzhou Strait and that in population LD is southward into the Gulf of Tonkin (Fig. 1). Although glacial rivers in the north of WY Ranges and rivers in Leizhou Peninsula experienced gene flow, the populations in the southern region of the WY Ranges were still genetically isolated from other mainland China populations. Thus, the gene flows between these two areas on two different sides of the WY Ranges might have been interrupted during glaciations and inter-glacial periods.

After the vicariance event of the WY Ranges formation, the ancestral populations in the northern region of Hainan Island and the mainland divergent into lineages I and II by another vicariance event, the Qiongzhou Strait (Figs 2 and 5b). The Qiongzhou Strait is a body of water that separates the Leizhou Peninsula to the north from Hainan Island to the south and connects the Gulf of Tonkin in the west to the South China Sea on the east. The strait is, on average, 30 km wide with a maximum water depth of approximately 120 m. During Pleistocene glaciations, the sea level dropped and exposed the present-day Qiongzhou Strait. At these times, migrants probably moved across the strait. However, the gene flow was interrupted by sinking of the strait during interglaciations. Chen et al. [7] proposed that populations of *G. hainanensis* migrated from one side to the other of the exposed Qiongzhou Strait and separated the populations in Hainan Island and the mainland into two highly divergent clades that were separated by the sea level rising. Moreover, based on the ichthyofauna, Hainan Island was identified as a unique subregion [1]. Thus, our study suggested that *G. orientalis* populations could exchange genes between on both sides of the strait when the mainland and island were in contact, but when the sea-level rose, the Qiongzhou Strait served as a phylogeographic break and disrupted the gene flow.

Furthermore, we found that lineage I was not only distributed in the southern region of the Yankai Mountains but also in the Pearl River and Zhejiang-Fujian subregions; lineage II was distributed in the northern region of Hainan Island and the Pearl River subregion (Fig. 2). Based on the results of S-DIVA (Fig. 2), our study suggested that when glaciation occurred again, lineages I and II dispersed northward from the northern region of Hainan Island (populations QH and BS) to the coastal populations in the Pearl River subregion and from the southern Yunkai Mountains (population CX) to the Zhejiang-Fujian subregion through exposed strait and continental shelves (Fig. 5c). Previous studies [2, 5, 7, 17, 47, 48] have proposed that during ice ages, the strait and continental shelves were largely above water and therefore, the coastal rivers may have become confluent with each other, an event which would have left an imprint in the composition of the fish fauna and the genetic structure. Thus, our study suggested that lineages I and II were admixed in coastal populations and then migrated from coastal populations to lower and upper tributaries of the Pearl River (scenario C) (Fig. 5d).

## Conclusion

The contemporary patterns of genetic diversity radically differed between mtDNA and microsatellite loci; however, congruent signals of population history were revealed by the approximate Bayesian computation approach (DIYABC). The discordances between mtDNA and microsatellite markers accounted for admixture events. The ancestral populations were distributed widely in the southern Yunkai Mountains, and the exposure and sinking of the strait and continental shelves contributed to the dispersion and differentiation of populations. Finally, *G. orientalis* is distributed widely in South China, excluding Taiwan Island. Moreover, the Wuzhishan and Yinggeling Mountain Ranges on Hainan Island and Qiongzhou Strait act as important phylogeographic breaks limiting migration between populations.

## Methods

### Ethics statement

The animal experiments were performed under an animal ethics approval granted by the Shanghai Ocean University. Our sampling procedures did not affect the survival of studies species.

### Sample collection and DNA isolation

In this study, specimens were collected from eight locations in the South China region (Fig. 1). These eight locations were divided into three subregions, Zhejiang-Fujian (JO and HA), Pearl River (CX, HY and JX) and Hainan Island (QH, BS and LD) based on ichthyofaunal classification by Li [1]. A total of 157 specimens of *G. orientalis* were collected (Fig. 1; Table 1). All specimens are lodged in the laboratory of Jin-Quan Yang, Key Laboratory of Exploration and Utilization of Aquatic Genetic Resources, Shanghai Ocean University. Specimens were collected from field sites with seines, fatally anesthetized with MS-222 (Sigma), and fixed and stored in 100 % ethanol. Genomic DNA was extracted from muscle tissue with a Genomic DNA Purification Kit (Gentra Systems, Valencia, CA).

### Mitochondrial DNA analysis

The mtDNA cyt b gene was amplified using polymerase chain reaction (PCR) with primers B-F (5‘-GCTCAGACTTTAACCGAGACCA AT-3’) [49] and H15915 (5‘-CTCCGATCTCCGGATTACAAGAC-3’) [50]. The D-loop DNA fragment was amplified with primers GODL-F (5‘-TAA AAGCATCG GTCTTGTAATC-3’) and GODL-R (5‘-CATGTGTAAGTTGAGTTAGAGCTG-3’), which were developed in our study. Each 50 μl PCR reaction mixture contained 5 ng template DNA, 5 μl 10x reaction buffer, 5 μl dNTP mix (10 mM), 5 pmol of each primer, and 2 U Taq polymerase (Promega, Madison, WI, USA). The PCR amplifications were performed using an Eppendorf Mastercycler (Eppendorf, Munich, Germany), with 1 cycle of denaturation at 94 °C for 3 min, 30 cycles of denaturation at 94 °C for 30 s, annealing at 52–56 °C for 30 s, and extension at 72 °C for 1 min, followed by a 72 °C extension for 10 min and 4 °C for storage. The PCR products were purified by electrophoresis in a 1.0 % agarose gel using 1x Tris–acetate–EDTA buffer. The gel was stained with ethidium bromide, and the desired DNA band was excised and eluted using an agarose gel purification kit (QIAGEN, Valencia, CA, USA). The products were sequenced using an ABI 377 automated sequencer (Applied Biosystems, Foster City, CA, USA). The chromatograms were evaluated with the CHROMAS software (Technelysium), and the sequences were manually edited using BIOEDIT 6.0.7 [51].

Nucleotide sequences were aligned with CLUSTALX 1.81 [52]. Levels of intra-population genetic diversity were estimated with indices of haplotype diversity (h) [53] and nucleotide diversity (θ_π_ and θ_ω_) [54] in DnaSP version 5.0 [55]. Comparing estimates of current (θ_π_) and historical (θ_ω_) genetic diversity provides insight into population dynamics over recent evolutionary history [56–58]. Patterns of geographical subdivision were estimated with F_ST_ hierarchically with DnaSP. The existence of phylogeographic structure was examined following Pons and Petit [59] by calculating two genetic differentiation indices, G_ST_ and N_ST_, in DnaSP. The substitution model used for the phylogenetic reconstructions was generated in jmodeltest 2 [60] based on Bayesian information criterion (BIC) scores. The HKY model (Hasegawa-Kishino-Yano) [61] was selected as the best model of nucleotide substitutions. *Garra imberba* (KM255666) [62] was used as an outgroup. The phylogenetic analyses were performed using maximum likelihood (ML), neighbor joining (NJ) and Bayesian inference (BI). The ML analysis used the programs in DAMBE v. 5.3.78 [63] and MEGA 6 [64]; the NJ analysis was performed in MEGA 6 with the Tamura & Nei model [65], similar to HKY. Bootstrapping was performed with 1000 replications. The BI analysis was implemented with MrBayes 3.0b4 [66]. The posterior probability values were used as support for the Bayesian topology. Log-likelihood stability was reached after approximately 500,000 generations, the first 350 trees were excluded as burn-in values, and the remaining trees were used to compute a 50 % majority rule consensus. The nucleotide divergence between samples was estimated with a Kimura two parameter (K2P) genetic distance in MEGA.

Several analyses in Arlequin version 3.5 [67] were used to investigate historical demographics including the haplotype network. This analysis was performed with a K2P distance and 20,000 permutations. Pairwise F_ST_ values and AMOVA (analysis of molecular variance) were used to partition variation among samples into within-population (F_ST_), within-group (F_SC_) and among-group (F_CT_) components. For the hierarchical analysis, populations were grouped according to several scenarios. Furthermore, to determine the possible diversification scenarios of *G. orientalis*, a statistical dispersal-vicariance analysis (S-DIVA), a program which complements DIVA, was employed to determine statistical support for ancestral range reconstructions [68, 69]. The tree file formats were generated by the program BEAST 1.8.0 [70] with 10^7^ MCMC steps and the first 10 % as burn-in. Each sampling population was defined by a range. The analysis was performed using the ‘maxareas = 4’ option. Additionally, our study attempted to estimate the time to the most recent common ancestor (T_MRCA_) based on the mtDNA data. However, the evolutionary rate of the mtDNA could not be estimated for *G. orientalis* because of a lack of a calibration point. A molecular clock was calibrated using the divergence rate of 2 % per million years, as previously postulated by Brown et al. [44] and Bermingham and Martin [45]. A stick clock model with Yule’s species tree prior was implemented in BEAST to estimate the T_MRCA_ values of the different lineages. The output was visualized in Tracer v1.6 [71] to determine that convergence and suitable effective sample sizes were achieved for all parameters.

The coalescent-based program IM (isolation with migration) [72] was used to estimate several directional migration rates. The parameters were scaled by the neutral mutation rate (μ): θ_A_, θ_1_ and θ_2_, are population mutation rates for the ancestral population and two daughter populations since divergence, where θ = N_e_μ and N_e_ is the effective population size; the time since population splitting (t), where t = Tμ and T is time in years; and the asymmetric migration rates between two populations (m_12_ and m_21_). IM analyses revealed unambiguous marginal posterior probability distributions of the parameters for all comparisons. The IM produced similar estimates for each run; parameter estimates fell within the 95 % highest posterior densities (HPD) for the parameter estimates from the other runs. The mode and 95 % HPD for estimates were used from the run producing the largest ESS for the estimates (ESS > 200).

### Microsatellite analysis

A total of 13 polymorphic microsatellite loci in *G. orientalis* (Gar 1–13) [37] were analyzed. The FSTAT version 2.9.3.2 program package [73] was used to calculate allele frequencies and estimate the expected (H_E_) and observed (H_O_) heterozygosity and the allelic richness (A_R_). GENEPOP web version 4.0.10 [74, 75] was used to test genotypic distributions for conformance to Hardy-Weinberg (HW) expectations, identify the loci in disequilibrium, and calculate F_ST_ values and the significance of genotypic differentiation between population pairs. This study also used a Bayesian procedure to infer population structure and estimate the number of genetically distinct populations using STRUCTURE v.2.2.3 [76]. Estimation of the number of sub-populations (*K*) was completed using 20 independent runs with *K* = 1–9 (assuming no prior population delineation information) at 100,000 MCMC repetitions combined with a 10,000 burn-in period. The analysis was performed with a burn-in of 2 × 10^6^ and 2 × 10^7^ iterations following the admixture model with prior population information. To obtain the most appropriate number of genetic groups in our dataset, we used the ad hoc statistic DK described by Evanno et al. [77]. This study selected the value of *K* best fitting our data using the log posterior probability of the data for a given K, Ln*Pr(X|K)* [78]*.* Finally, this study additionally estimated population level admixture by calculating the mean of the individual admixture coefficients. To explore relationships among the populations, Nei’s D_A_ distance [79] between all pairs of populations was calculated, and a dendrogram was constructed using the neighbor-joining (NJ) method [80] with bootstrap values calculated by POPULATIONS ver. 1.2.28 (http://bioinformatics.org/∼tryphon/populations/).

### Population history

To reconstruct the unknown history of divergences among these eight populations, we performed approximated Bayesian computations (ABC) on our mtDNA and microsatellite data set with the software DIYABC v.2.0 [81]. The DIYABC program allows for the comparison of different historical scenarios involving population divergence, admixture and population size changes and then infers demographic and historical parameters under the best-supported scenario. These scenarios are as follows:

In the first scenario (null hypothesis, Fig. 4a), all populations diverged simultaneously. In scenario B (mtDNA phylogenetic model, Fig. 4b), according to the phylogenetic analysis of mtDNA (Fig. 2), populations have diverged in two successive events: the population in the southern region of Hainan Island (LD) diverged first, the populations in the Zhejiang-Fujian subregion (JO and HA) and the northern region of Hainan Island diverged later (BS and QH), and finally, there has been an admixture event between the populations in the northern region of Hainan Island and the Zhejiang-Fujian subregion generated an admixed populations in the Pearl River subregion (CX, JX and HY). In scenario C (mtDNA S-DIVA model, Fig. 4c), based on the S-DIVA analysis of mtDNA (Fig. 2), the ancestral populations are distributed in the Hainan Island (LD, BS and QH) and southern Pearl River (CX) subregions, and populations have diverged in two successive events: the population in the southern region of Hainan Island (LD) diverged first, and the populations in the southern region of the Pearl River (CX) and the northern region of Hainan Island (BS and QH) diverged later. Finally, the ancestral population of CX dispersed northward to the Zhejiang-Fujian subregion (JO and HA), and then, there was an admixture event between the populations in the northern region of Hainan Island and the ancestral population CX, giving rise to admixed populations in the Pearl River subregion (CX, JX and HY). Scenario D (microsatellite phylogenetic model, Fig. 4d) was reconstructed based on the phylogenetic analysis of microsatellites (Fig. 3c). Scenario E (microsatellite STRUCTURE model, Fig. 4e) was reconstructed based on the combined results of a STRUCTURE analysis of microsatellites (Fig. 3a) and the NJ tree’s visualized genetic distance among the inferred clusters (Fig. 3b).

Our study used three data sets, mtDNA, microsatellites and mtDNA + microsatellites, to examine these five scenarios (Fig. 4). The ABC analyses are based on the simulation of 5,000,000 genetic data sets under all the scenarios. All scenarios were compared using a logistic regression approach, and parameter estimation was performed for the scenario with the highest posterior probability only.

### Availability of supporting data

The sequence dataset generated herein is available in the GenBank repository with Accession numbers KR698422-KR698486 and KR698391-KR 698421.
